# Supplementary material for: p53 pulse modulation differentially regulates target gene promoters to regulate cell fate decisions
Source: Mol Syst Biol. 2019 Sep 26;15(9):e8685. doi: 10.15252/msb.20188685 (PMC6761572; doi:10.15252/msb.20188685)
Supplement: Supplementary file 1 — Expanded View Figures PDF [file MSB-15-e8685-s001.pdf]

Expanded View Figures

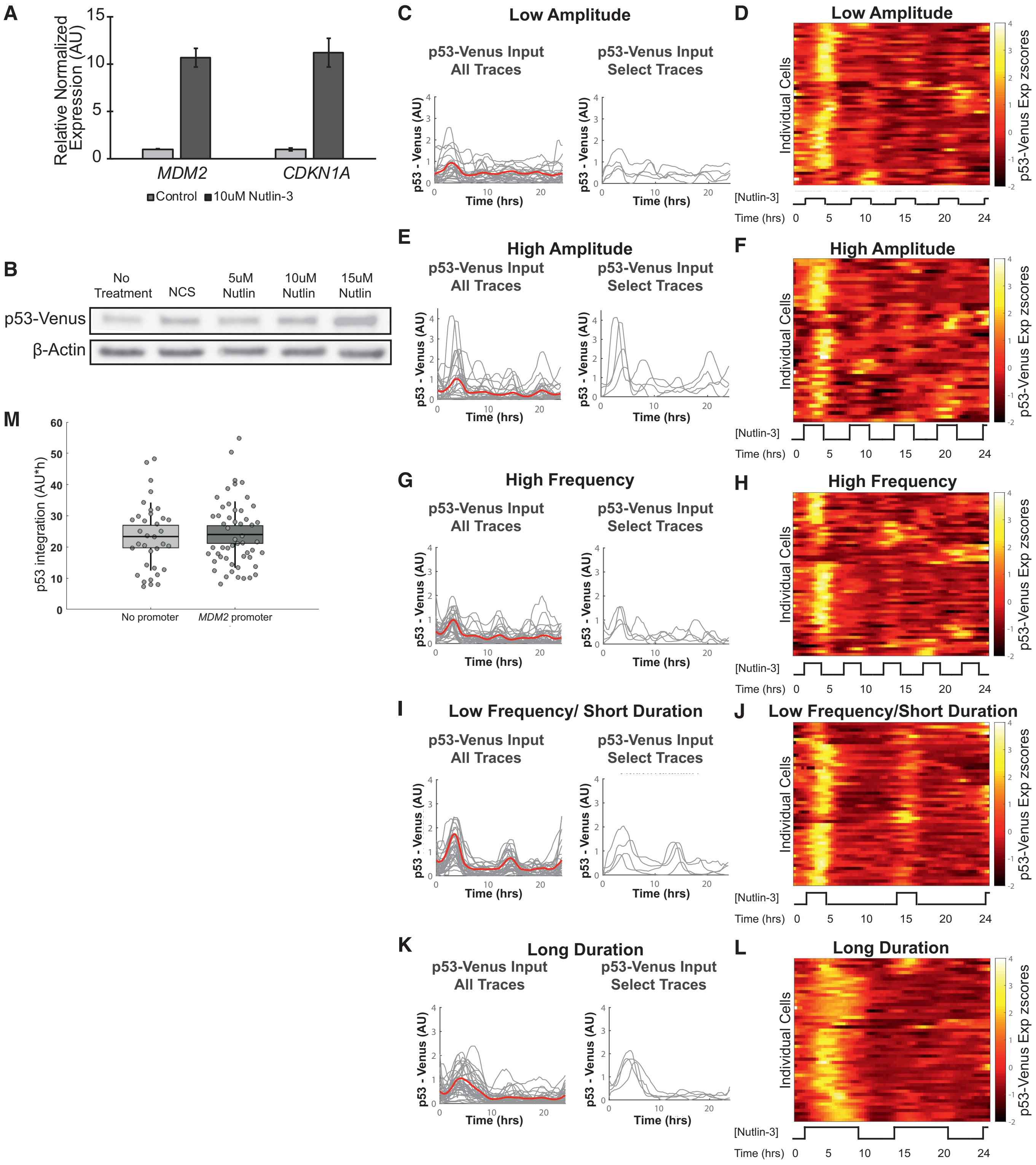

Figure EV1.

 © 2019 The Authors

Figure EV1. Characterization of p53-Venus expression dynamics.

A mRNA levels for endogenous *MDM2* and *CDKN1A* in response to treatment with or without 10 μM Nutlin-3. Error bars = SEM ($n = 2$).

B Western blot of p53-Venus levels in the *MDM2* promoter reporter cell line untreated or treated with 400 ng/μl neocarzinostatin (NCS), 5 μM Nutlin-3, 10 μM Nutlin-3, or 15 μM Nutlin-3 for 3 h.

C–L p53-Venus expression in response to the low-amplitude (C, D), high-amplitude (E, F), high-frequency (G, H), low-frequency/short-duration (I, J), and long-duration (K, L) Nutlin-3 dosing regimens. Single-cell traces (gray) and the mean (red) are shown in (C, E, G, I, K) for p53-Venus expression in response to each Nutlin-3 regimen. Heat maps (D, F, H, J, L) shown as alternative representations of all traces as shown in (E). N = at least 45 cells per condition.

M p53-Venus levels integrated over 24 h for cell lines with or without the *MDM2* promoter reporter exposed to Nutlin-3 treatment. Line = mean, box = SD, bar = 95% confidence interval (N = at least 45 cells).

Source data are available online for this figure.

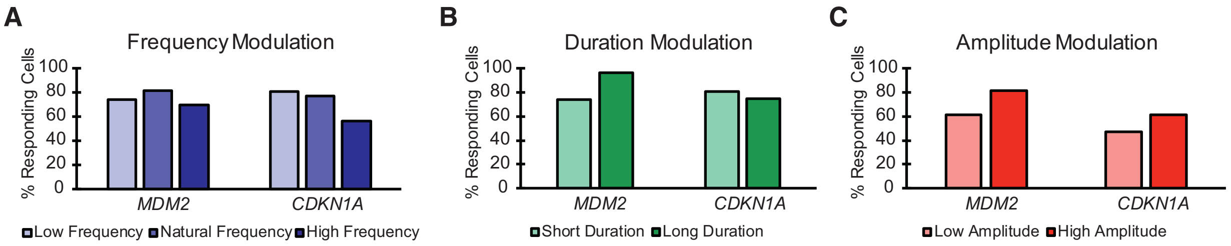

Figure EV2. Percentage of responding cells for p53 pulse modulations.

A–C Percentage of cells showing an increase of mCherry induction from the indicated promoters in response to p53 frequency (A), duration (B), or amplitude (C) modulation. Data for Fig EV2A also shown in Fig 5H.

Source data are available online for this figure.

Figure EV3. *K*-means clustering analysis reveals distinct target promoter activation profiles for p53 pulse modulations.

A–L Partition of individual *MDM2* (pink) and *CDKN1A* (purple) promoter-mCherry activation traces into four clusters based on *k*-means clustering analysis for low-amplitude (A, B), high-amplitude (C, D), long-duration (E, F), low-frequency (G, H), natural-frequency (I, J), and high-frequency (K, L) p53 pulse modulations.

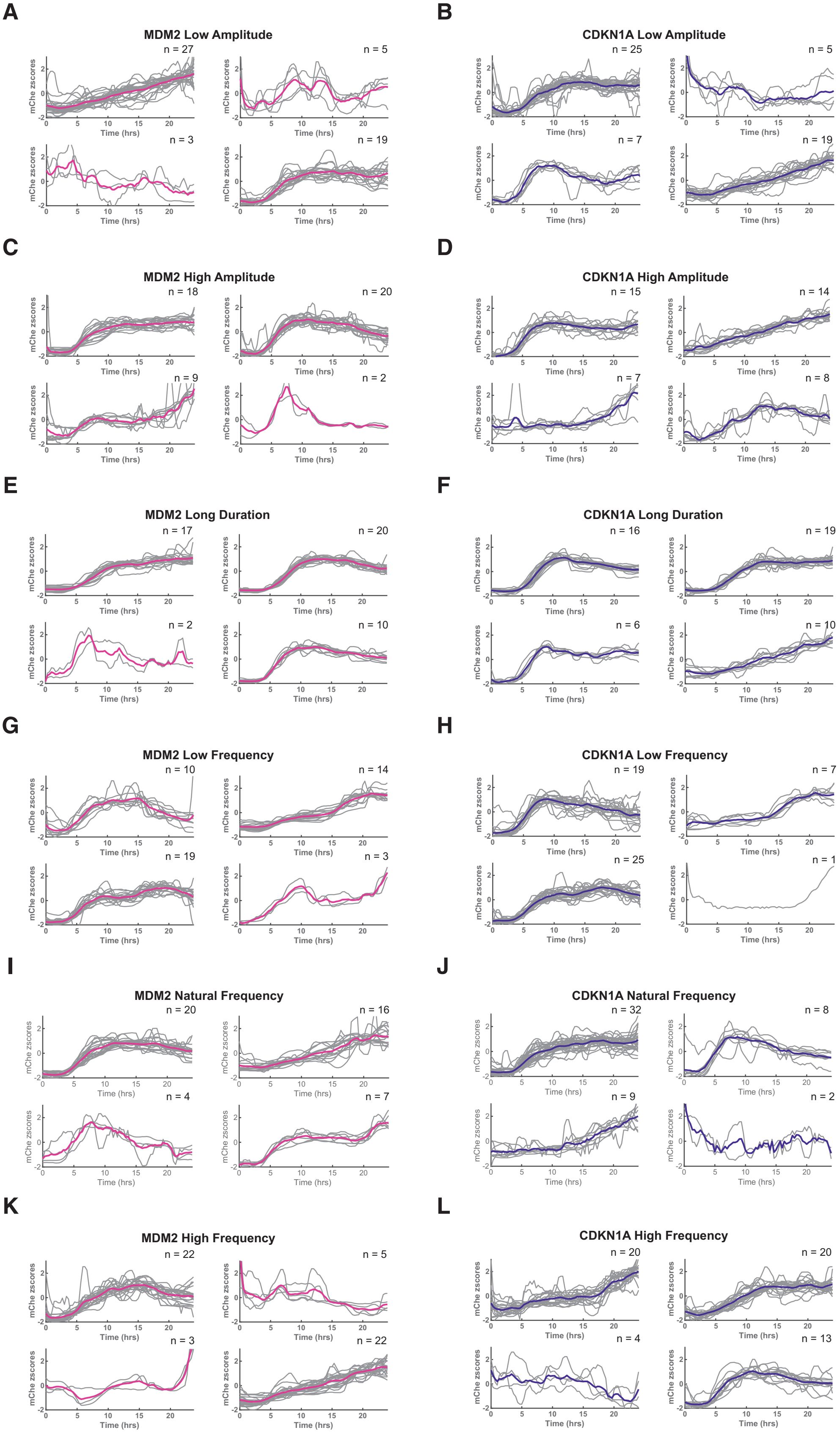

Figure EV3.

 © 2019 The Authors

 © 2019 The Authors

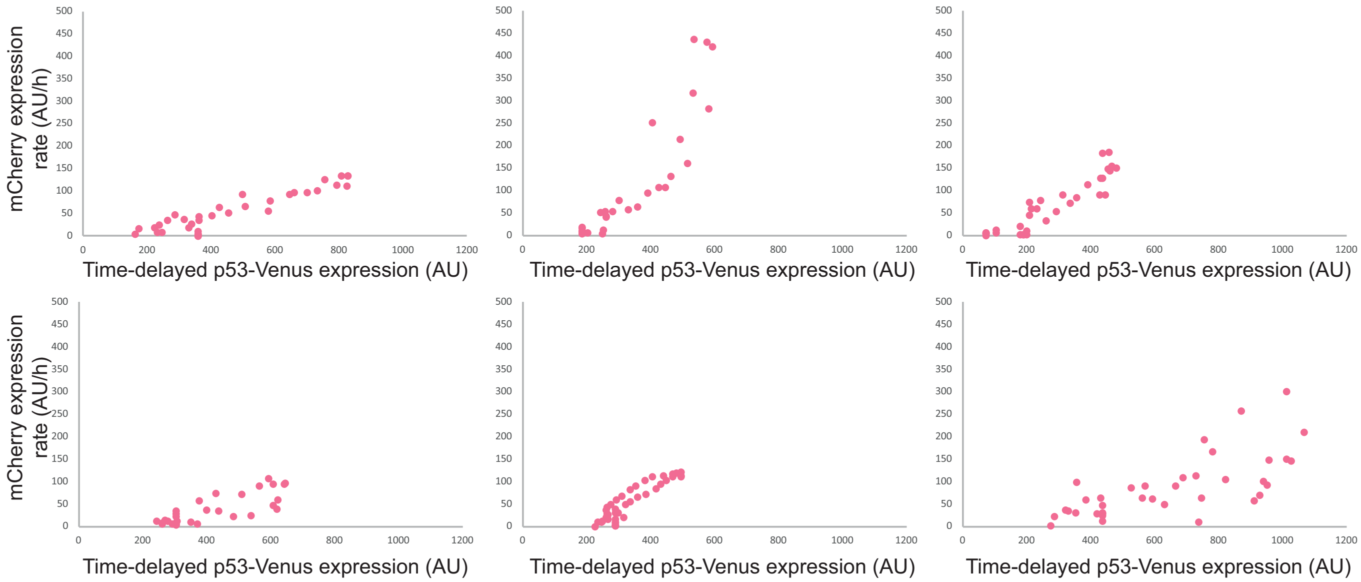

Figure EV4. Individual cells show highly variable promoter responses to p53 expression.

Dose–response curves representing the rate of mCherry expression from the *MDM2* promoter (pink dots) as a function of total p53 levels in six representative single cells in the first 15-h response to the long-duration Nutlin-3 dosing regimen.

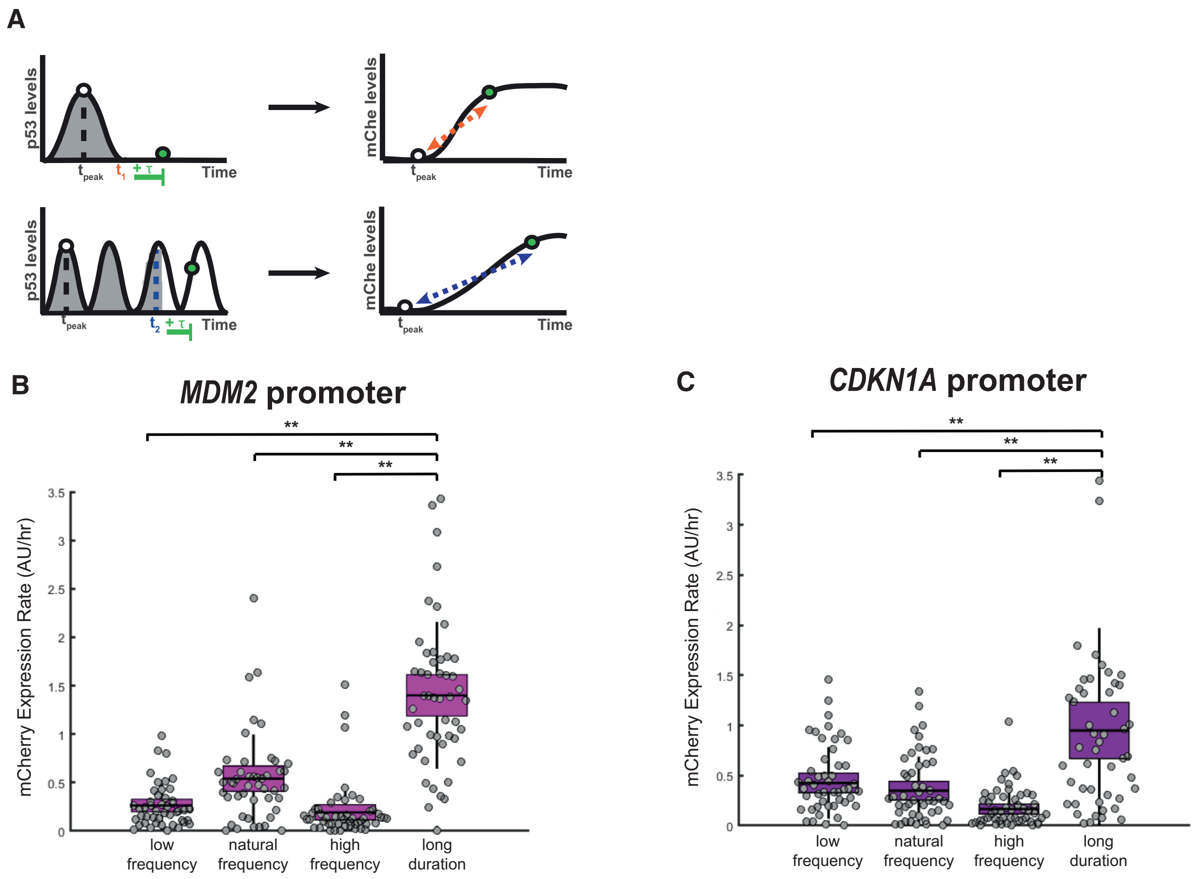

Figure EV5.

Figure EV5. Target promoters show different sensitivities toward p53 temporal modulation.

A Schematic for comparing the rate of target promoter activation when p53 is at comparable cumulative levels in response to duration- and frequency-modulated p53 inputs. Rates were calculated between the time of the first p53 pulse peak (t_{peak}) and the time at which the cumulative p53 levels are equal plus an additional 5.5 h ($t_X + \tau$), where τ = 5.5 h is the natural p53 pulse period.

B, C Activation rates of the *MDM2* (B) and *CDKN1A* (C) promoters at times of equivalent cumulative p53 levels in response to temporal pulse modulations. Line = mean, box = SD, bar = 95% confidence interval (N = at least 45 cells). $^{**}P < 0.01$, two-sample t-test.

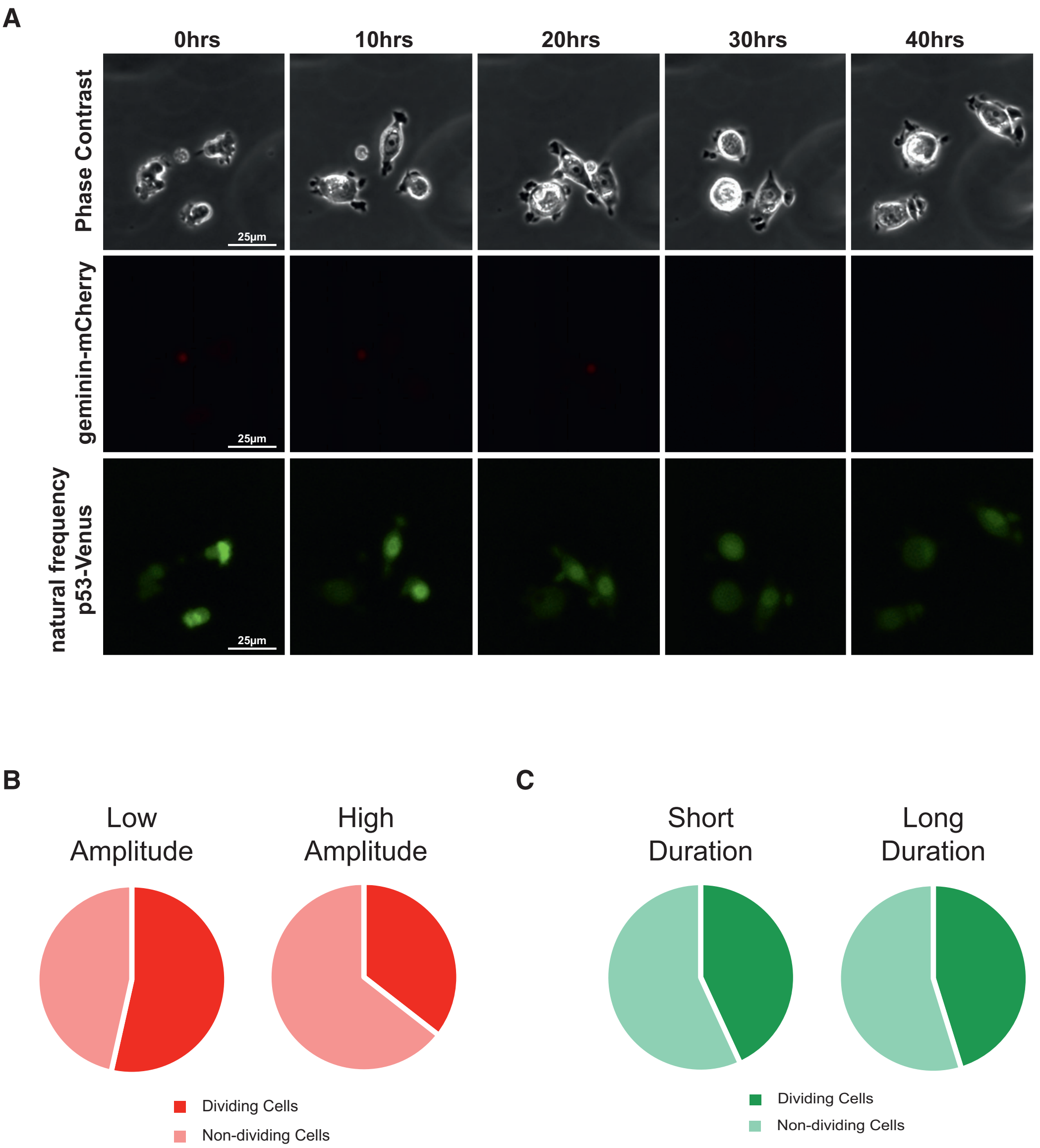

Figure EV6. Geminin-mCherry reporter tracks the level of cell cycle arrest in response to p53 amplitude and duration modulation.

A Representative phase contrast, red fluorescence (indicating geminin-mCherry levels), and yellow fluorescence (indicating p53-Venus levels) images of cells in response to the "natural dynamics" Nutlin-3 dosing regimen over 40 h.

B, C Percentage of cells undergoing cell division within the 40 h of imaging in response to amplitude modulation (B) or duration modulation (C) Nutlin-3 dosing regimens. N = at least 102 cells per condition.

© 2019 The Authors

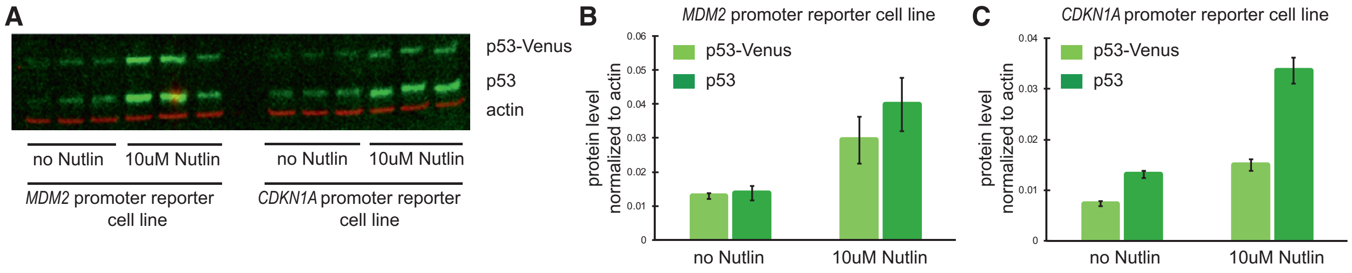

Figure EV7. Quantification of p53 and p53-Venus levels in the MDM2 promoter and CDKN1A promoter clonal cell lines.

A Western blot against p53 and actin in the MDM2 and CDKN1A promoter reporter cell lines in basal conditions or in response to treatment with 10 μM Nutlin-3 for 3 h. Biological triplicate experiments are shown.

B, C Quantification of the p53 and p53-Venus levels normalized to actin from (A). Error bars = SEM (n = 3).
